# Diagnostic accuracy of RISK6 assay in childhood pulmonary TB

**DOI:** 10.5588/ijtldopen.24.0603

**Published:** 2025-07-09

**Authors:** S. Pouzol, M.K.M. Uddin, A. Islam, T. Alam, M.S. Jabin, S. Banu, J. Hoffmann

**Affiliations:** ^1^ Medical and Expertise Division, Fondation Mérieux, Lyon, France;; ^2^ Infectious Diseases Division, icddr,b, Dhaka-1212, Bangladesh;; ^3^ Nutrition Research Division, icddr,b, Dhaka-1212, Bangladesh.

**Keywords:** tuberculosis, TB diagnostic, triage test, pediatric, biomarker, cost-effectiveness

## Abstract

**BACKGROUND:**

The WHO recently updated the target product profile (TPP) for TB identification at the peripheral level to guide test development.

**METHODS:**

We conducted a prospective diagnostic accuracy study among outpatients under 15 years old with presumed pulmonary TB at the International Centre for Diarrheal Disease Research, Bangladesh (icddr,b) Dhaka Hospital. We evaluated the accuracy of the RISK6 assay and performed a cost-per-TB case identification analysis.

**RESULTS:**

Of 365 enrolled children, 68 had microbiologically confirmed TB, 94 had unconfirmed TB and 203 were unlikely to have TB. RISK6 did not meet the TPP for TB diagnosis in children but in presumptive pulmonary TB individuals ≤ 12 months, the assay outperformed (32/42) both the Mantoux test (7/42) and chest X-ray (11/42) in correctly referring individuals for confirmatory testing. RISK6, alone or in combination with Mantoux test, was the most cost-effective strategy for identifying and confirming TB, with a cost as low as $US132.00 in children > 12 months.

**CONCLUSION:**

Although RISK6 did not meet the WHO TPP for TB diagnosis, it shows promise as a triage test, especially for children ≤ 12 months, and could serve as a decision-support tool in integrated treatment algorithms recommended by the WHO.

In 2023, an estimated 1.3 million children under 15 years of age developed TB, representing 12% of the global burden, yet only 57% of these children were notified to the WHO.^1^ In Bangladesh, an estimated 34,000 children (9.0% of all TB cases) developed TB in 2023, but only 12,100 childhood TB cases were notified to the WHO (4% of all notified TB cases), leaving 21,900 children undiagnosed and/or untreated.^1^

Diagnosing pulmonary TB (PTB) in children necessitates an integrated approach, combining a comprehensive clinical assessment (evaluating signs and symptoms such as fever, malnutrition, respiratory distress, weight loss, and persistent cough) with investigation of the child’s exposure to a TB patient and confirmatory diagnostic testing. The clinical presentation of TB in children often mimics other common childhood illnesses such as pneumonia, HIV-associated lung disease, and malnutrition, making clinical diagnosis difficult.^2^ With better sensitivity than symptom screening, chest X-ray (CXR) is an important tool for the diagnosis of childhood TB in both PTB and extra-pulmonary TB, and proves effective when placed early in screening and triaging algorithms.^3^ Despite inability to confirm TB disease and potential cross-reactivity with Bacille Calmette-Guérin (BCG) vaccine, the tuberculin skin test (TST) remains an option for confirming *Mycobacterium tuberculosis* (*M.tb*) infection in children under five years in high endemic settings.^4^ The paucibacillary nature of TB in children and the difficulty in collecting adequate respiratory specimens,^5^ make acid-fast bacilli microscopy, culture, and molecular tests suboptimal for microbiological confirmation.^6^ Recent studies and reviews,^7–10^ have been conducted to assess the current status of biomarkers that can be used to diagnose *M.tb* infection, asymptomatic and symptomatic TB. Many of them were derived from profiling the transcriptional response of host peripheral blood immune cells, such as RISK6,^11^ a 6-gene blood transcriptomic signature for TB diagnosis and treatment monitoring. The RISK6 signature has been evaluated in a multi-country study with adult pulmonary TB and demonstrated performance meeting the WHO target product profile (TPP) benchmarks^12^ as a sputum-free screening and triage test. However, the performance of RISK6 in children remains unexplored making the cost and optimal placement in clinical algorithms unclear.

This study aimed to assess RISK6 assay’s diagnostic accuracy in children, evaluate the signature’s performance as a triage or diagnostic test, and compare the cost-effectiveness of various strategies to enhance TB case identification in children.

## METHODS

### Study design and participants

This prospective diagnostic accuracy study, conducted from March 2022 to April 2023, enrolled children under 15 years with presumptive PTB who presented as inpatients or outpatients at Dhaka Hospital of the International Centre for Diarrheal Disease Research, Bangladesh (icddr,b). Children were screened and eligible for inclusion if they exhibited two or more of the following symptoms or signs: persistent, non-remitting cough lasting for more than 2 weeks, and/or persistent documented fever (axillary temperature >38°C) for more than 2 weeks, and/or documented weight loss or failure to gain weight over the past 3 months or severe malnutrition, and/or fatigue, reduced playfulness and decreased activity. Malnutrition was classified according to the WHO child growth standards,^13^ using mid-upper arm circumference (MUAC), weight-for-length, or clinical signs of bilateral pitting oedema for children aged 6–59 months as well as body mass index (BMI) for children aged 5 to 15 years^14^ (see Supplementary Data). Written informed consent was obtained from parents/legal guardians, and additional assent was obtained from children older than 7 years.

Eligible participants who provided consent were consecutively enrolled and underwent a comprehensive TB workup, including demographic data collection, symptoms assessment and medical history using a standardized case report form (CRF). CXRs were also performed, and a radiologist used a dedicated CRF based on the National Tuberculosis Guidelines for Children,^15^ to document CXR findings and abnormalities. If the CXR interpretation was ‘Abnormal compatible with TB’, the opinions of at least two expert reviewers or paediatric consultants were obtained before making any decisions. Children not diagnosed with TB were followed up by phone. Children undergoing anti-tuberculosis treatment were clinically evaluated two months after treatment initiation and again upon treatment completion.

### Laboratory procedures and diagnostic classification

Induced sputum, stool and blood samples were collected from each of the participants at enrolment (Supplementary Data). Induced sputa were cultured in Löwenstein-Jensen (LJ)-medium and tested with Xpert MTB/RIF Ultra (Xpert) for microbiological confirmation. Stool samples were tested with a second Xpert. For each patient, 3.0 mL of blood was collected directly in Tempus Blood RNA tubes (Thermo Fischer Scientific, USA) for RISK6 assay, a 6-gene transcriptomic signature for diagnosis of TB disease, performed blind to clinical assessment and microbiological results. The RISK6 score was calculated from the relative expression of six genes^11^ using extracted mRNA (protocol in Supplementary Data). Each gene’s expression cycle threshold (Ct) values were measured in replicates. The mean Ct values of each pair of replicates were calculated for the targeted genes and combined to compute a score ranging from 0 to 1 (1 being the highest probability of PTB).^12^ Participants were assigned to diagnostic categories based on the clinical case definitions of intrathoracic TB in children.^2^ The primary outcome was to confirm or rule-out TB disease, with participants categorized as ‘TB confirmed, ‘TB unconfirmed’, or ‘TB unlikely’. Classification was based on the microbiological test results, clinical signs and symptoms, radiological findings, TB contact history, *M.tb* infection status, and clinical response to treatment, including data on visit attendance, treatment adherence, symptom resolution, and anthropometric changes over time. Two different reference standards were used to estimate diagnostic accuracy.^16^ The microbiological reference standard (MRS) classified children as confirmed TB (positive) only if they had a positive Xpert or culture result. All other children were classified as unlikely TB (negative). The composite clinical reference standard (CRS) incorporated clinical diagnosis and children classified as confirmed and unconfirmed TB were considered as positive, while those classified as unlikely TB were considered negative for the analysis.

### Cost per TB case identification analysis

A model was developed and parameterised with data from the study to compare different triage and confirmatory strategies in a cohort of 10,000 children with presumptive TB seeking care in a peripheral setting (Supplementary Data). The analysis estimated the cost per TB case identified for each alternative diagnostic strategy, using only the direct costs identified (reagents, tests, medical consultation).

### Statistical analysis

Test performance was assessed using the MRS and the CRS as reference standards. The Area Under the Curve (AUC) and its 95% confidence intervals (95% CI) were used to determine the accuracy of the RISK6 assay. AUCs between groups were compared using the DeLong method and Youden's J index was used to identify the optimal probability cut-off for the assay. Data were cleaned and analysed using R version 4.3. The pROC package (including the roc.test function) was used for receiver operating characteristic (ROC) curve analysis. Summary statistics (median and interquartile range [IQR]) were calculated using the Finalfit package. Differences in proportions were compared by the χ^2^ test and Mann–Whitney test was used for comparison of data that were not normally distributed. A probability of less than 0.05 was considered statistically significant. The individuals were stratified by age due to the significant difference (p < 0.01) in the RISK6 score at the 12-month threshold in discriminating between confirmed TB and unlikely participants.

### Sample size

Due to the lack of prior evaluation of RISK6 in a child population, a precise sample size calculation was not possible. However, with an estimated 15.2% TB prevalence in children with presumed TB^17^ and an annual screening volume of 350–400 presumptive children, we projected to recruit 60 confirmed TB patients and 340 unconfirmed TB patients. Assuming an AUC of 65.0%, the samples size was projected to provide 96.7% statistical power.

### Ethics approval

The protocol was approved by the Institutional Review Board (IRB) of icddr,b and recorded under a protocol reference number, PR-21102.

## RESULTS

Among 1979 children screened at Dhaka Hospital, 465 (23.5%) presented with signs and symptoms suggestive of PTB. Of these, 85 (18.3%) did not provide consent. From the 380 consenting participants, 8 (2.1%) did not receive the TST and 7 (1.8%) could not be tested with RISK6 assays (Figure). Among the 365 assessable participants, 68 (18.6%) had microbiologically confirmed TB, 94 (25.8%) had unconfirmed TB and 203 (55.6%) were classified as unlikely TB patients. The median age of the study population was 10.1 months (IQR 5.9–17.0) with 306 of the 365 (83.8%) being under 2 years (Table 1); 226 (61.9%) were male and 296 (81.1%) were malnourished at enrolment. Out of 68 confirmed cases, 67 (98.5%) tested positive with Xpert either from induced sputum (n=42) or stool (n=34) samples, and 2 were culture-positive but only one was microbiologically confirmed. The median RISK6 scores were significantly different (p < 0.005) between children under 12 months of age and older children, with higher median RISK6 score in confirmed TB cases (0.60 vs. 0.47) and unconfirmed TB cases (0.48 vs. 0.37).

**Figure.**
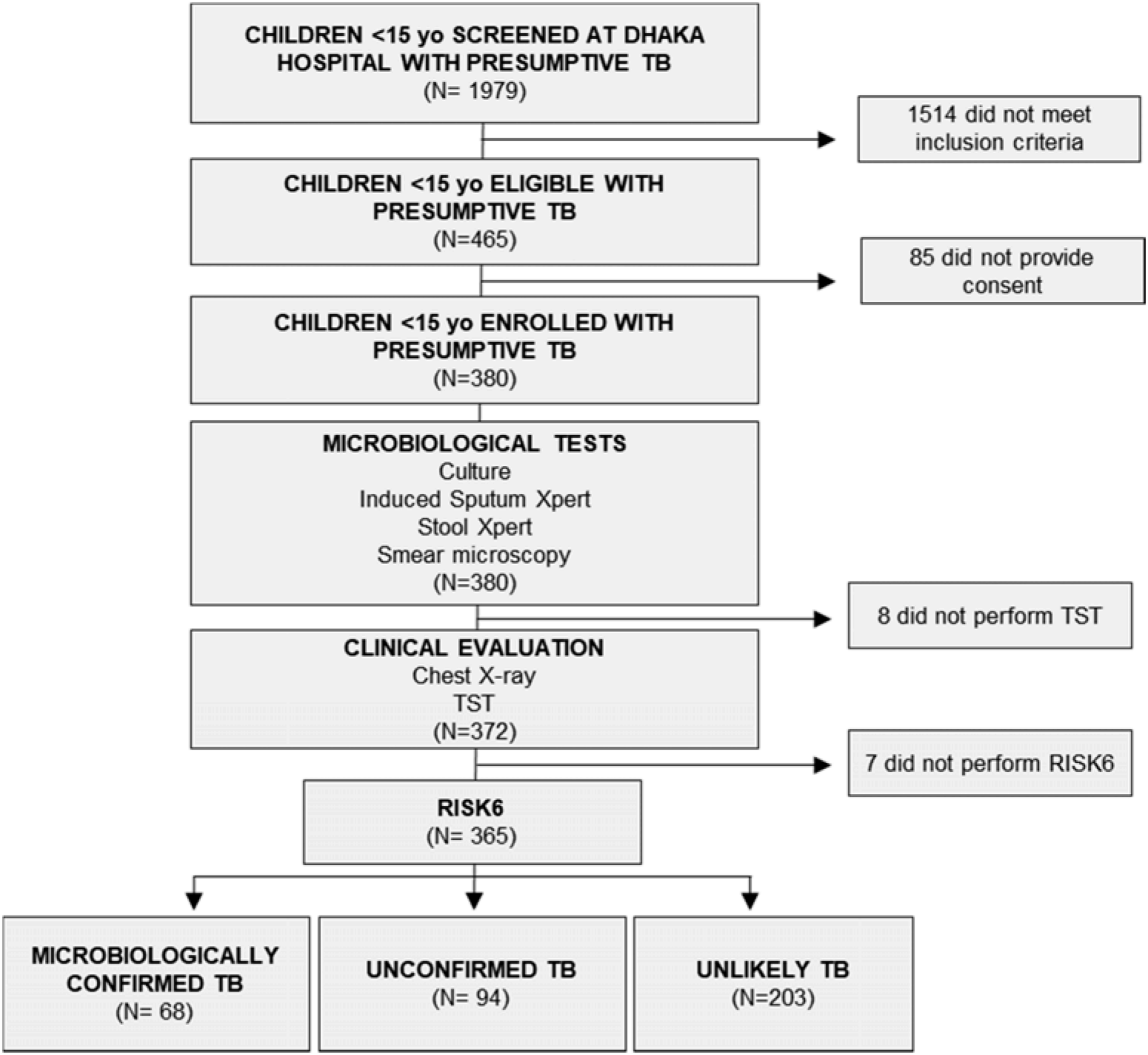
Flow diagram of participants the study. All children with defined clinical case definition (see Methods section) were included in the analysis. TST = tuberculin skkin test; Xpert = Xpert MTB/RIF Ultra diagnostic test; RISK =: six whole blood gene transcriptomic signature; N = number.

**Table 1.** Baseline sociodemographic and clinical characteristics of presumptive TB patients stratified by clinical diagnosis classification.

			Confirmed TB	Unconfirmed TB	Unlikely TB	Total	p
			(n=68)	(n=94)	(n=203)	(n=365)	
**Demographics**							
	Age [Median (IQR)]	Months	9.9 [5.6 to 14.8]	11.1 [7.0 to 19.8]	9.4 [5.5 to 16.1]	10.1 [5.9 to 17.0]	0.286
	Sex	Female	23 (33.8%)	37 (39.4%)	79 (38.9%)	139 (38.1%)	0.723
		Male	45 (66.2%)	57 (60.6%)	124 (61.1%)	226 (61.9%)	
	Dwelling	Rural	44 (64.7%)	41 (43.6%)	107 (52.7%)	192 (52.6%)	0.030
		Urban	24 (35.3%)	53 (56.4%)	96 (47.3%)	173 (47.4%)	
**Comorbidities**							
	Malnutrition	Severe	47 (69.1%)	53 (56.4%)	126 (62.1%)	226 (61.9%)	0.414
		Moderate	9 (13.2%)	19 (20.2%)	42 (20.7%)	70 (19.2%)	
		No	12 (17.6%)	22 (23.4%)	35 (17.2%)	69 (18.9%)	
	HIV	No	68 (100.0%)	94 (100.0%)	202 (99.5%)	364 (99.7%)	0.670
		Yes			1 (0.5%)	1 (0.3%)	
**Clinical symptoms at enrolment**							
	Cough >2 weeks	No	16 (24.2%)	20 (25.3%)	39 (21.7%)	75 (23.1%)	0.788
		Yes	50 (75.8%)	59 (74.7%)	141 (78.3%)	250 (76.9%)	
	Tachypnea	No	52 (76.5%)	82 (87.2%)	154 (75.9%)	288 (78.9%)	0.071
		Yes	16 (23.5%)	12 (12.8%)	49 (24.1%)	77 (21.1%)	
**Risk factors**							
	Exposure	No	59 (86.8%)	58 (61.7%)	187 (92.1%)	306 (83.8%)	<0.001
		Yes	9 (13.2%)	36 (38.3%)	16 (7.9%)	59 (16.2%)	
	PreviousTB	No	66 (98.5%)	91 (96.8%)	201 (99.5%)	358 (98.6%)	0.179
		Yes	1 (1.5%)	3 (3.2%)	1 (0.5%)	5 (1.4%)	
**TB-related clinical, immunological and microbiological findings**							
	TST	Negative	53 (77.9%)	52 (55.3%)	200 (98.5%)	305 (83.6%)	<0.001
		Positive	15 (22.1%)	42 (44.7%)	3 (1.5%)	60 (16.4%)	
	Chest X-ray consistent with tuberculosis	Negative	51 (75.0%)	61 (64.9%)	203 (100.0%)	322 (88.2%)	<0.001
		Positive	17 (25.0%)	33 (35.1%)	0 (0.0%)	43 (11.8%)	
	Xpert Induced Sputum	Negative	26 (38.2%)	94 (100.0%)	203 (100.0%)	323 (88.5%)	<0.001
		Positive	42 (61.8%)			42 (11.5%)	
	Xpert Stool	Negative	34 (50.0%)	94 (100.0%)	203 (100.0%)	331 (90.7%)	<0.001
		Positive	34 (50.0%)			34 (9.3%)	
	Culture	Negative	66 (97.1%)	92 (100.0%)	202 (100.0%)	360 (99.4%)	0.013
		Positive	2 (2.9%)			2 (0.6%)	
	RISK6 Score [Median (IQR)]	≤12 months	0.60 [0.50–0.81]	0.48 [0.33–0.75]	0.45 [0.33–0.69]	0.49 [0.28–0.60]	0.0073
		>12 months	0.47 [0.35–0.60]	0.37 [0.28–0.48]	0.45 [0.28–0.63]		
**Outcome**							
	Death	No	65 (95.6%)	93 (98.9%)	202 (99.5%)	360 (98.6%)	0.053
		Yes	3 (4.4%)	1 (1.1%)	1 (0.5%)	5 (1.4%)	

TST = tuberculin skin test; Xpert = Xpert MTB/RIF Ultra diagnostic test; IQR = interquartile range. Data are n (%) or median [IQR].

Regardless of the reference standard used, the RISK6 test showed a higher AUC in children ≤ 12 months than older children (Table 2). In the ≤ 12 months group, the RISK6 assay achieved an AUC of 67.2% (95% CI 58.1-76.0), with a sensitivity of 78.6% and a specificity of 56.1% using MRS. With CRS, the AUC was 58.5% (95% CI 51.1-66.0), with a sensitivity of 59.6% and specificity of 57.7%.

**Table 2.** RISK6 diagnostic accuracy performance as a function of reference standards used, stratified by age group.

	Threshold	AUC (%)	95% CI (%)	Sp (%)	Se (%)	NPV (%)	PPV (%)
MRS ≤ 12 months (n=172)	0.485	67.1	58.1–76.0	56.1	78.6	89.0	36.7
MRS > 12 months (n=99)	0.345	52.1	39.8–64.4	37.0	84.6	87.1	32.4
CRS ≤ 12 months (n=229)	0.495	58.5	51.1–66.0	57.7	59.6	65.2	51.7
CRS > 12 months (n=136)	0.426	53.4	43.6–63.2	56.1	55.6	59.4	52.2

MRS = microbiological reference standard; CRS = composite reference standard; RISK6 = six whole blood gene transcriptomic signature; AUC = area under the curve; CI = confidence interval; Se = sensitivity; Sp = specificity; NPV = negative predictive value; PPV = positive predictive value. TN = true negative; TP = true positive; FN = false negative; FP = false positive. Sensitivity = TP/(TP+FN); Specificity = TN/(TN+FP); NPV = TN/(TN+FN); PPV = TP/(TP+FP).

The performance of TST and CXR, evaluated against the CRS, was similar across all age groups, with AUCs ranging from 64.3% to 68.5% for each test individually, and up to 79.6% when combined (Table 3). Although RISK6 demonstrated lower overall performance, it outperformed TST and CXR in referring confirmed TB cases for further testing, particularly in children ≤ 12 months (76.2% vs. 16.7% and 26.2%, respectively). An iterative strategy combining TST or CXR followed by RISK6 testing on the remaining negative cases increased both the proportion of confirmed TB cases identified (88.1%) and the number of individuals referred for confirmatory testing.

**Table 3.** Evaluation of test performance using CRS as reference standard (triage strategies).

		Threshold	AUC (%)	95% CI (%)	Sp (%)	Se(%)	TN(n)	TP(n)	FN(n)	FP(n)	Positive (TP+FP) Fraction		Negative (TN+FN) Fraction	
											To confirmatory testing (n)	TB Confirmed (%)	To confirmatory testing (n)	TB Confirmed (%)
≤ 12 months	RISK6 Assay	0.495	58.5	51.1–66.0	57.7	59.6	75	59	40	55	114	76.2	115	23.8
	CXR	—	66.2	61.5–70.8	100	32.3	130	32	67	0	32	26.2	197	73.8
	CXR then RISK6*	0.505	66.5	60.4–72.6	59.2	73.7	77	73	26	53	126	81.0	103	19.0
	TST	—	65.8	61.1–70.5	99.2	32.3	129	32	67	1	33	16.7	196	83.8
	TST then RISK6*	0.455	65.3	59.4–71.1	50.8	79.8	66	79	20	64	143	88.1	86	11.9
	TST+CXR	—	79.4	74.5–84.3	99.2	59.6	129	59	40	1	60	38.1	169	61.9
														
> 12 months	RISK6 Assay	0.595	53.4	43.6–63.2	56.2	55.6	41	35	28	32	67	46.2	69	53.8
	CXR	—	64.3	58.7–69.9	100	28.6	73	18	45	0	18	23.2	118	76.8
	CXR then RISK6 *	0.595	54.1	46.0–62.2	68.5	39.7	50	25	38	23	48	38.5	88	61.5
	TST	—	68.5	62.1–74.8	97.3	39.7	71	25	38	2	27	30.8	109	69.2
	TST then RISK6*	0.575	57.7	49.3–66.0	63.0	52.4	46	33	30	27	60	53.8	76	46.2
	TST+CXR	—	79.6	73.3–85.9	97.3	61.9	71	39	24	2	41	50.0	95	50.0

* 2-Step strategy. TST = tuberculin skin test; CXR = chest X-ray; RISK6 = six whole blood gene transcriptomic signature; AUC = area under the curve; CI = confidence interval; Se = sensitivity; Sp = specificity; TN = true negative; TP = true positive; FN = false negative; FP = false positive; sensitivity = TP/(TP+FN); specificity = TN/(TN+FP).

In the modelling cohort (Table 4), the most cost-effective strategies per TB case identified (relative to no test) were CXR + RISK6 ($US159.00) for children ≤ 12 months and TST + RISK6 ($US132.00) for older children, using sputum and stool samples for confirmatory testing. Modelling a strategy meeting WHO TPP requirements resulted in a reduced cost per TB case identified, reaching $US116.00 when using TST + RISK6 for children over 1 year of age, followed by blood sample confirmatory testing.

**Table 4.** Cost ($US) per TB case identified.

		Cost ($US) per TB case identified					
		Xpert sputum	Xpert stool	Both Xpert (any positive)	WHO TPP sputum	WHO TPP stool	WHO TPP blood
≤ 12 months	RISK6 assay	271	274	197	195	181	179
	CXR	452	472	301	341	329	327
	CXR + RISK6	220	222	159	159	147	145
	TST test	443	452	312	324	306	302
	TST + RISK6	326	322	247	229	207	203
	CXR and/or TST test	398	410	276	295	280	277
> 12 months	RISK6 assay	387	559	303	308	286	282
	CXR	440	659	315	368	356	354
	CXR + RISK6	241	352	184	195	183	181
	TST test	230	332	181	183	170	167
	TST + RISK6	164	235	132	129	118	116
	CXR and/or TST test	259	379	198	209	197	195

TST = tuberculin skin test; CXR: chest X-ray; RISK6: six whole blood gene transcriptomic signature; TPP = target product profile.

## DISCUSSION

Among the numerous studies exploring the use of transcriptomic signatures for TB diagnosis, only a few focused exclusively on TB disease in children, with most concentrating on distinguishing children infected with *M.tb* from healthy uninfected controls.^18^ In this study, the RISK6 signature was evaluated among children with presumed PTB and demonstrated interesting diagnostic performance among children under 12 months with an AUC of 67.1%, 78.6% sensitivity and 56.1% specificity. However, the assay did not meet the optimal WHO TPP for a non-sputum, near-point of care identification test at the peripheral level which required 75.0% sensitivity and 98.0% specificity.^19^ The reason(s) for the poorer performance observed among children older than 12 months remains unclear. No significant differences in RISK6 scores were observed based on the sample type used for confirmation testing, TST result or malnutrition status. There was also no correlation between the Xpert burden levels (trace, very low, or low) and the RISK6 scores. Notably, for Xpert results other than ‘trace’, the RISK6 scores were at least 0.52 in children under 12 months (and ≥ 0.81 in the two culture-positive cases), while it ranged from 0.35 to 0.57 in older children. In addition, of the children who died during the study (n=6), only one was over one year old (13 months). This might suggest that individuals over 12 months in our study had milder TB disease and individuals under 12 months might have had more severe TB. Nevertheless, our results align with a multicentre study evaluating the Cepheid *Mycobacterium tuberculosis* Host Response prototype cartridge,^16^ which reported an AUC of 66.0% in children ≤ 12 months.

In high disease burden settings, triage is a key strategy for identifying individuals who may have TB and require further evaluation for diagnosis. However, no optimal triage test for TB currently exists. The most common strategy, CXR after symptom screening, is limited by infrastructure and training requirements. Although the WHO does not recommend TST for screening TB disease in children, it remains useful when combined with symptom screening in areas where CXR cannot be performed routinely. False-negative TST results can occur due to factors such as recent infection (8–10 weeks post-exposure) or malnutrition (present in 81.1% of this study’s population), which could explain the low proportion of TST-positive tests in the confirmed TB group of this study (15/53 overall, including 7/33 among children under 12 months).

Although RISK6 has limitations in distinguishing between confirmed and unconfirmed TB cases (Supplementary Data) and exhibits low specificity, it outperforms TST and CXR in referring confirmed TB cases for further testing, increasing the total number of individuals requiring confirmation. Given the high morbidity and mortality rates associated with TB in children, the risk of missing a diagnosis is generally deemed more critical than the risk of false positives and unnecessary treatment. While a $US 4 WHO TPP-compliant test would be desirable, large-scale implementation may result in costs comparable to existing methods (although increasing sensitivity beyond 75% could reduce overall TB case identification costs). Beyond its demonstrated cost-effectiveness compared to TST and CXR, a portable, affordable (target price of $US 10), battery-operated RISK6 testing device using a finger-prick blood sample will soon be available. Re-evaluation of this development may make RISK6 an attractive option as a companion test for triage in low-resource, decentralized settings especially when combined with TST.

Culture remains the gold standard method for TB diagnostic accuracy study despite its lower and heterogeneous yield. This study reported a very low rate of positive cultures (2.9%) in confirmed cases, necessitating alternative reference standards for performance analysis. Microbiological confirmation of TB cases was therefore, based on both Xpert sputum and stool results, leading to a low positive inter-assay concordance (9/34) and a higher TB incidence rate (23.3%) in the study population compared to the initial estimate of 15.2%. Combining Xpert results from different samples complicates comparison and may introduce bias in evaluating RISK6 performance. Lastly, this single-centre study, conducted in setting with low HIV prevalence and high malnutrition rate may limit the generalizability of the results.

## CONCLUSION

This study addresses the challenges of diagnosing PTB in children and highlights the potential of RISK6 as a triage tool for identifying individuals who require confirmatory testing. RISK6, alone or in combination, offers an interesting and lower cost alternative to WHO-hypothetical test for TB management in children, particularly in areas where CXR cannot be deployed. Further studies are required to replicate these findings and additional evaluation is also necessary to assess the added value of RISK6 as a decision-support tool for healthcare workers using WHO-integrated treatment decision algorithms for paediatric TB.^20^

## Supplementary Material


