# Supplementary material for: Diagnostic accuracy of RISK6 assay in childhood pulmonary TB
Source: IJTLD Open. 2025 Jul 9;2(7):397–403. doi: 10.5588/ijtldopen.24.0603 (PMC12248410; doi:10.5588/ijtldopen.24.0603)
Supplement: Supplementary file 1 [file ijtldopen24-0603_supplementarydata1.pdf]

Indicator	Measure	Cut-off
Severe wasting (*2*)	Weight-for-height (*1*)	< -3 SD
Severe wasting (*2*)	MUAC	< 115 mm
Bilateral oedema (*3*)	Clinical sign	

1 Based on WHO Standards (www.who.int/childgrowth/standards)
2,3 Independent indicators of SAM that require urgent action

Figure S1. Diagnostic criteria for Severe Acute Malnutrition in children aged 6-60 months.

From World Health Organization, United Nations Children's Fund (UNICEF). WHO child growth standards and the identification of severe acute malnutrition in infants and children : joint statement by the World Health Organization and the United Nations Children's Fund. 2009;11.
Available from https://iris.who.int/handle/10665/44129

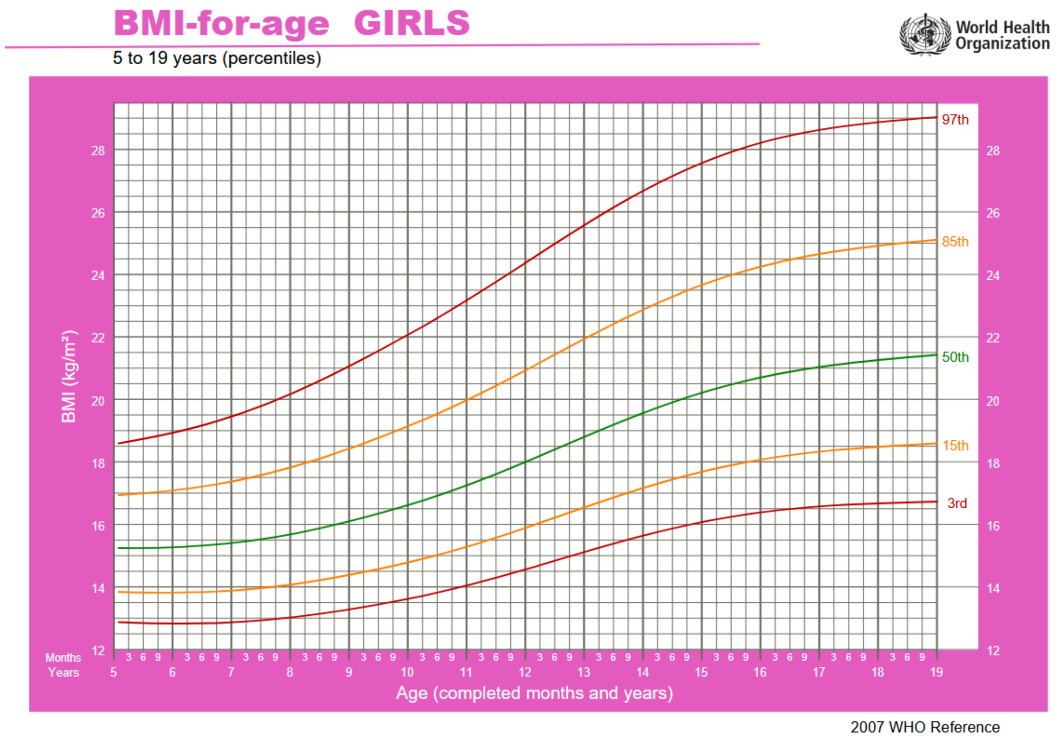

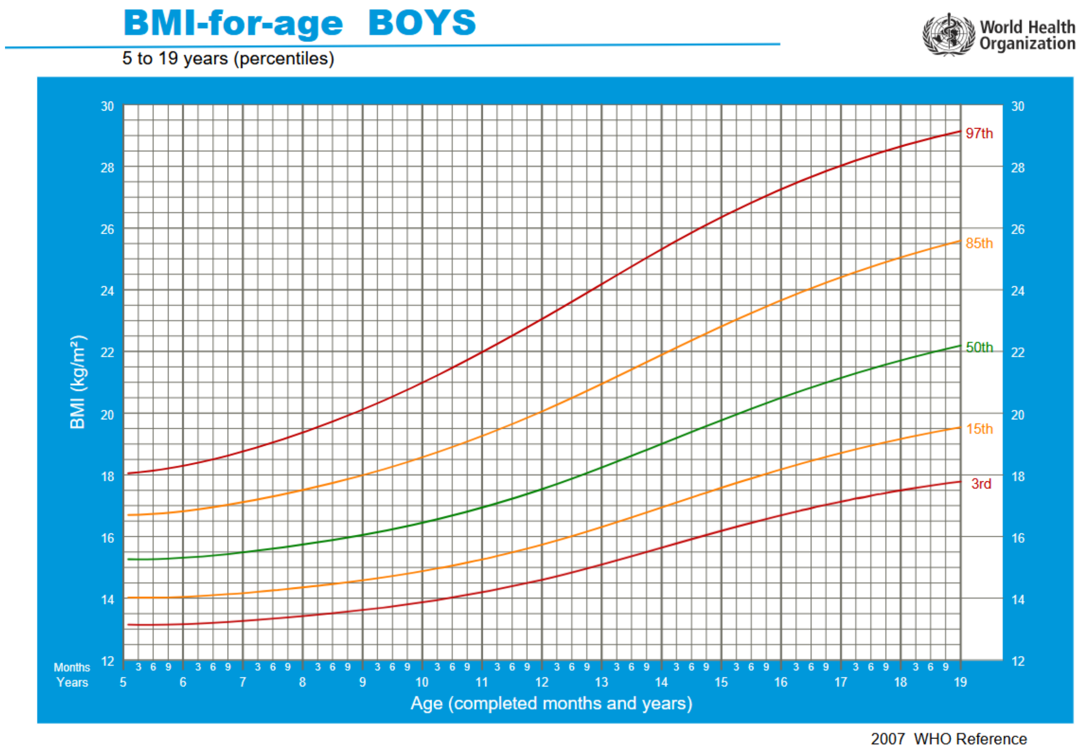

Figure S2. BMI reference value for Malnutrition in children aged 5-15 years.

Adapted from de Onis M, et al. Development of a WHO growth reference for school-aged children and adolescents. Bull World Health Organ. 2007;85(9):660–7. Available from https://www.who.int/tools/growth-reference-data-for-5to19-years/indicators/bmi-for-age

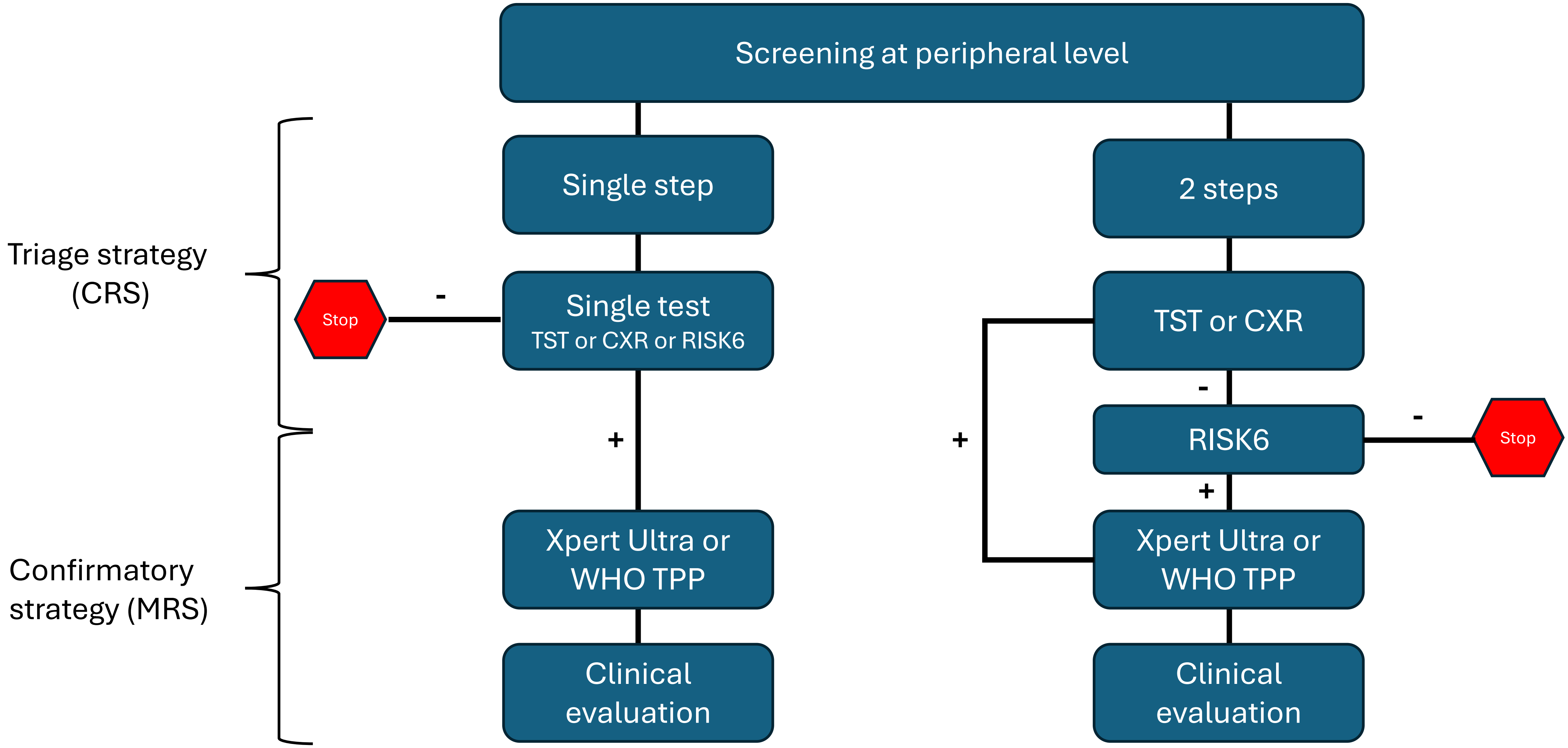

Figure S3. Cost per TB case identification algorithm

CRS: Clinical Reference Standard; MRS: Microbiological Reference Standard CXR: Chest X-Ray; TST: Tuberculin Skin Test; WHO: World Health Organization; TPP: Target Product profile. WHO TPP = hypothetical test meeting WHO Target Product Profile requirement

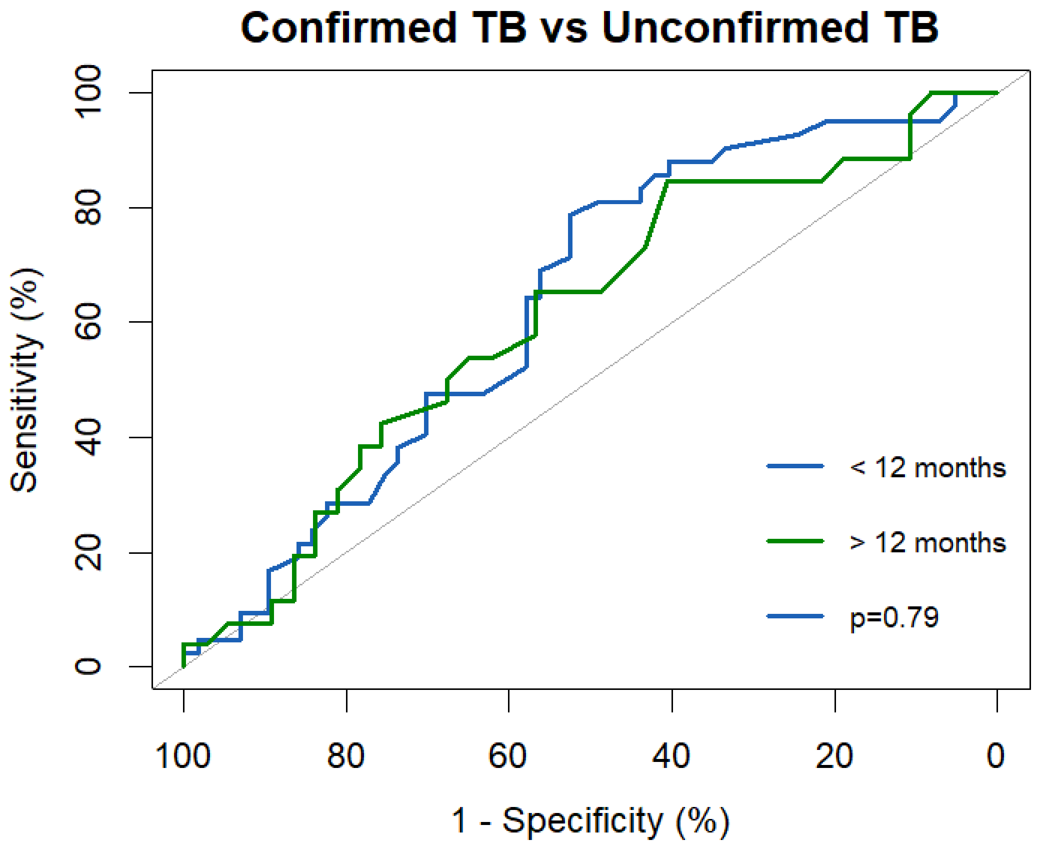

Figure S4. Receiver Operating Characteristic (ROC) curves depicting the performance of RISK6, stratified by age group, to discriminate between confirmed TB cases and unconfirmed TB cases (TB treatment as reference standard).

Table S1. Test performance results using CRS and distribution of individuals according to clinical case classification.

	**Test performance according Study Results (CRS)**						**Distribution (n) in Positive Fraction (FP+TP)**		
	Specificity (%)	Sensitivity (%)	TN (n)	TP (n)	FN (n)	FP (n)	Confirmed TB	Unconfirmed TB	Unlikely TB
≤12 months									
TST test	99.2	32.3	129	32	67	1	7	25	1
CXR	100	32.3	130	32	67	0	11	21	0
RISK6 Assay	57.7	59.6	75	59	40	55	32	27	55
RISK6 Assay after TST	50.8	79.8	66	79	20	64	37	42	64
RISK6 Assay after CXR	59.2	73.7	77	73	26	53	34	39	53
TST+CXR	99.2	59.6	129	59	40	1	16	43	1
>12 months									
TST test	97.3	39.7	71	25	38	2	8	17	2
CXR	100	28.6	73	18	45	0	6	12	0
RISK6 Assay	56.2	55.6	41	35	28	32	12	23	32
RISK6 Assay after TST	63.0	52.4	46	33	30	27	14	19	27
RISK6 Assay after CXR	68.5	39.7	50	25	38	23	10	15	23
TST+CXR	97.3	61.9	71	39	24	2	13	26	2

CRS: Clinical Reference Standard; CXR: Chest X-Ray; TST: Tuberculin Skin Test; TN: True Negative; TP: True Positive; FN: False Negative; FP: False Positive.

Table S2. Test performance results in hypothetical cohort using CRS and distribution of individuals according to clinical case classification.

	TB Prevalence	Hypothetical Cohort (CRS)						Distribution (n) in fraction referred to confirmation			
		Specificity (%)	Sensitivity (%)	TN (n)	TP (n)	FN (n)	FP (n)	Confirmed TB	Unconfirmed TB	Unlikely TB	Total
≤12 months											
TST test	43.2	99.2	32.3	5,631	1,396	2,927	45	305	1,091	45	1,441
CXR		100	32.3	5,677	1,396	2,927	0	480	916	0	1,396
RISK6 Assay		57.7	59.6	3,276	2,577	1,747	2,401	1,398	1,179	2,401	4,978
RISK6 Assay after TST*	36.4	50.8	79.8	2,935	3,370	853	2,842	843	2,528	2,842	6,212
RISK6 Assay after CXR*	38.7	59.2	73.7	3,420	3,112	1,111	2,357	1,449	1,663	2,357	5,469
TST+CXR	43.2	99.2	59.6	5,632	2,577	1,746	45	699	1,878	45	2,622
>12 months											
TST test	46.3	97.3	39.7	5,223	1,839	2,793	145	588	1,251	145	1,984
CXR	46.3	100	28.6	5,368	1,325	3,308	0	442	883	0	1,325
RISK6 Assay	46.3	56.2	55.6	3,017	2,576	2,057	2,351	883	1,693	2,351	4,927
RISK6 Assay after TST*	46.3	63.0	52.4	3,382	2,427	2,205	1,986	1,030	1,397	1,986	4,413
RISK6 Assay after CXR*	46.3	68.5	39.7	3,731	1,839	2,793	1,637	736	1,103	1,637	3,476
TST+CXR	46.3	97.3	61.9	5,223	2,867	1,765	145	956	1,911	145	3,012

CRS: Clinical Reference Standard; CXR: Chest X-Ray; TST: Tuberculin Skin Test; TN: True Negative; TP: True Positive; FN: False Negative; FP: False Positive.

* = Total also includes individuals with positive TST or CXR (2-Step strategy)

Table S3. Distribution of confirmed TB individuals after confirmatory testing (MRS as reference standard).

	TB prevalence in Positive Fraction	Individuals who underwent confirmation (n)	Distribution of confirmed TB individuals after confirmatory testing (MRS)				
			Expected Confirmed TB (n)	Confirmed TB identified with Xpert Sputum (n)	Confirmed TB identified with Xpert Stool (n)	Confirmed TB identified with Any Xpert (n)	Confirmed TB identified with WHO TPP test (n)
≤12 months							
TST test	21.2%	1,441	305	181	167	298	229
CXR	34.4%	1,396	480	284	263	468	360
RISK6 Assay	28.1%	4,978	1,398	827	766	1,364	1,048
RISK6 Assay after TST*	13.6%	6,212	843	499	462	822	632
RISK6 Assay after CXR*	26.5%	5,469	1,449	858	794	1,414	1,085
TST+CXR	26.7%	2,622	699	414	383	682	524
>12 months							
TST test	29.7%	1,984	588	385	249	588	441
CXR	33.3%	1,325	442	289	187	442	331
RISK6 Assay	17.9%	4,927	883	578	373	883	663
RISK6 Assay after TST*	23.3%	4,413	1,030	673	436	1,030	772
RISK6 Assay after CXR*	21.2%	3,476	736	481	311	736	552
TST+CXR	31.7%	3,012	956	625	404	956	717

MRS: Microbiological Reference Standard; CXR: Chest X-Ray; TST: Tuberculin Skin Test; Xpert: Xpert MTB/Rif Ultra; WHO: World Health Organization; TPP: Target Product profile. WHO TPP = hypothetical test meeting WHO Target Product Profile requirement

Table S4. Cost Approach

	Unit price ($US)
TST	1.7
CXR	6.6
Xpert*	7.97
RISK6†	10
WHO TPP	4
Medical consultation	4
Induced sputum sampling	4
Blood drop sampling	0.5
Stool sampling	1

CXR: Chest X-ray; TST: Tuberculin Skin Test; Xpert: Xpert MTB/Rif Ultra; WHO: World Health Organization; TPP: Target Product profile. WHO TPP = hypothetical test meeting WHO Target Product Profile requirement
*Global fund source; †: target price

Table S5. Triage Cost Calculation

	Sample size (n)	Triage Cost ($US)		
		Medical consultation	Test	Sampling
≤12 months				
TST test	10,000	40,000	17,000	
CXR	10,000	40,000	66,000	
RISK6 Assay	10,000	40,000	100,000	5,000
RISK6 after TST	10,000 + 6,212	40,000	79,120	3,106
RISK6 after CXR	10,000 + 5,469	40,000	120,690	2,735
CXR and/or TST	10,000	40,000	83,000	
>12 months				
TST test	10,000	40,000	17,000	
CXR	10,000	40,000	66,000	
RISK6 Assay	10,000	40,000	100,000	5,000
RISK6 after TST	10,000 + 4,413	40,000	61,130	2,207
RISK6 after CXR	10,000 + 3,476	40,000	100,760	1,738
CXR and/or TST	10,000	40,000	83,000	

CXR: Chest X-ray; TST: Tuberculin Skin Test; Xpert: Xpert MTB/Rif Ultra;

Table S6. Confirmation Cost Calculation

	Individuals referred for confirmation (n)	Confirmation Cost ($US)						
		Medical Consultation	Lab Testing			Sampling		
			Xpert	Any Xpert	WHO TPP	Sputum	Stool	Blood
≤12 months								
TST test	1,441	5,764	11,485	22,970	5,764	5,764	1,441	720.5
CXR	1,396	5,584	11,126	22,252	5,584	5,584	1,396	698
RISK6 Assay	4,978	19,912	39,675	79,349	19,912	19,912	4,978	2,489
RISK6 after TST	6,212	24,848	49,510	99,019	24,848	24,848	6,212	3,106
RISK6 after CXR	5,469	21,876	43,588	87,176	21,876	21,876	5,469	2734.5
CXR and/or TST	2,622	10,488	20,897	41,795	10,488	10,488	2,622	1,311
>12 months								
TST test	1,984	7,936	15,812	31,625	7,936	7,936	1,984	992
CXR	1,325	5,300	10,560	21,121	5,300	5,300	1,325	662.5
RISK6 Assay	4,927	19,708	39,268	78,536	19,708	19,708	4,927	2463.5
RISK6 after TST	4,413	17,652	35,172	70,343	17,652	17,652	4,413	2206.5
RISK6 after CXR	3,476	1,738	27,704	55,407	13,904	13,904	3,476	1,738
CXR and/or TST	3,012	3,012	24,006	48,011	12,048	12,048	3,012	1,506

CXR: Chest X-ray; TST: Tuberculin Skin Test; Xpert: Xpert MTB/Rif Ultra;

Table S7. Incremental Cost Calculation

	Incremental Cost ($US)					
	Xpert			WHO TPP		
	Sputum	Stool	Any Xpert	Sputum	Stool	Any Xpert
≤12 months						
TST test	80,013	75,690	92,939	74,292	69,969	69,249
CXR	128,294	124,106	140,816	122,752	118,564	117,866
RISK6 Assay	224,499	209,565	269,151	204,736	189,802	187,313
RISK6 after TST	221,432	202,796	277,153	196,770	178,134	175,028
RISK6 after CXR	250,764	234,357	299,821	229,053	212,646	209,911
CXR and/or TST	164,873	157,007	188,393	154,464	146,598	145,287
>12 months						
TST test	88,684	82,732	106,481	80,808	74,856	73,864
CXR	127,160	123,185	139,046	121,900	117,925	117,263
RISK6 Assay	223,684	208,903	267,879	204,124	189,343	186,880
RISK6 after TST	173,812	160,573	213,397	156,293	143,054	140,847
RISK6 after CXR	185,844	175,416	217,023	172,044	161,616	159,878
CXR and/or TST	162,066	153,030	189,083	150,108	141,072	139,566

CXR: Chest X-ray; TST: Tuberculin Skin Test; Xpert: Xpert MTB/Rif Ultra; WHO: World Health Organization; TPP: Target Product profile. WHO TPP = hypothetical test meeting WHO Target Product Profile requirement

Table S8. Evaluation of RISK6 performance using TB treatment as reference standard, stratified by age group.

	AUC (%)	95% CI	Sp (%)	Se (%)	NPV (%)	PPV (%)	TN	TP	FN	FP
≤ 12 months	63.6	52.6-74.6	52.6	78.6	76.9	55.0	30	33	9	27
> 12 months	61.1	46.8-75.3	40.5	84.6	78.9	50.0	15	22	4	22

AUC: Area Under the Curve; CI: Confidence Interval; Se: Sensitivity; Sp: Specificity; Sensitivity: TP/(TP+FN); Specificity: TN/(TN+FP); NPV: Negative Predictive Value: TN/(TN+FN); PPV: Positive Predictive Value: TP/(TP+FP); TP: True Positive; TN: True Negative; FP: False Positive; FN: False Negative.

Table S9. Evaluation test performance using MRS as reference standard, stratified by age group.

	Threshold	AUC (%)	95% CI (%)	Sp (%)	Se (%)	NPV (%)	PPV (%)	TN	TP	FN	FP
≤ 12 months											
RISK6 Assay	0.485	67.1	58.1-76.0	56.2	78.6	89.0	36.7	73	33	9	57
Xpert induced sputum assay	/	79.8	72.2-87.3	100	59.2	88.4	100	130	25	17	0
Xpert stool assay	/	77.4	69.8-85.0	100	54.8	87.2	100	130	23	19	0
CXR	/	63.1	56.4-69.8	56.4	69.8	80.7	26.2	130	11	31	0
> 12 months											
RISK6 Assay	0.345	52.1	39.8-64.4	37.0	84.6	87.1	32.4	27	22	4	46
Xpert induced sputum assay	/	82.7	73.4-92.0	100	65.4	89.0	100	73	17	9	0
Xpert stool assay	/	71.2	61.5-80.8	100	42.3	82.8	42.3	72	11	15	0
CXR	/	61.5	53.3-69.8	100	23.1	78.5	100	73	6	20	0

CXR: Chest X-ray; RISK6: six whole blood gene transcriptomic signature; AUC: Area Under the Curve; CI: Confidence Interval; Se: Sensitivity; Sp: Specificity; TN: True Negative; TP: True Positive; FN: False Negative; FP: False Positive; NPV: Negative Predictive Value; PPV: Positive Predictive Value. Sensitivity: TP/(TP+FN); Specificity: TN/(TN+FP); NPV: TN/(TN+FN); PPV: TP/(TP+FP).

Table S10. Evaluation of Xpert Ultra performance using CRS as reference standard, stratified by age group.

	Threshold	AUC (%)	95% CI (%)	Sp (%)	Se (%)	NPV (%)	PPV (%)	TN	TP	FN	FP
≤ 12 months											
Xpert induced sputum assay	/	62.6	58.3-66.9	100	25.3	63.7	100	130	25	74	0
Xpert stool assay	/	61.6	57.4-65.8	100	23.2	63.1	100	130	23	76	0
> 12 months											
Xpert induced sputum assay	/	63.5	58.0-69.0	100	27.0	61.3	100	73	17	46	0
Xpert stool assay	/	58.7	54.0-63.5	100	17.5	58.1	100	72	11	52	0

AUC: Area Under the Curve; CI: Confidence Interval; Se: Sensitivity; Sp: Specificity; TN: True Negative; TP: True Positive; FN: False Negative; FP: False Positive; NPV: Negative Predictive Value; PPV: Positive Predictive Value. Sensitivity: TP/(TP+FN); Specificity: TN/(TN+FP); NPV: TN/(TN+FN); PPV: TP/(TP+FP).

Supplementary Document 1 – Malnutrition classification

Malnutrition was classified according to WHO child growth standards using mid-upper arm circumference (MUAC), weight-for-length, or clinical signs of bilateral pitting oedema for children aged 6-59 months (Figure S1) as well as body mass index (BMI) for children aged 5 to 15 years (Figure S2). Severe malnutrition was defined as a z-score below 3 Standard Deviations (SD), while moderate malnutrition was defined as a z-score between 1 and 3 SDs.

Supplementary Document 2 – Samples collection

Blood sample collection

At enrolment, 3 mL blood was collected directly into Tempus Blood RNA tubes (Thermo Fischer Scientific, USA) containing 6 mL of Tempus reagent, shaken vigorously, and stored at − 80 °C. An additional 1 mL of blood was collected in a lithium heparin tube which was transferred to a BioSample card (Ahlstrom-Munksjo, France) and stored at − 20 °C for biobanking purpose.

Induced sputum collection

Induced sputum was collected from children using a standardized protocol. Patients were advised to refrain from eating or drinking for at least 4 hours before the procedure. Nebulization with 5 mL of 3% sodium chloride solution was initiated to induce sputum production. Subsequently, a nebulizer containing 1.25 mL of salbutamol solution and 2.5 mL of 0.9% sodium chloride solution was administered until fully inhaled. Patients were encouraged to cough frequently during the nebulization process. Induced sputum was collected in a sterile container while the patient coughed. For children unable to expectorate sputum, a soft-headed tube was gently inserted through the nose or mouth, and sputum was collected using negative pressure After collection, 5 mL of 0.9% sodium chloride solution was added to the specimen to facilitate processing.

Stool sample collection

Caregivers (most often a parent) of the children were given sterile stool containers and instructed to place a portion of fresh stool specimen into the container (half full, approximately 10 g). Stool samples were processed with in-house laboratory-developed methods described previously (1).

Sample Transport

All samples were sent for processing and storage to the Mycobacteriology Laboratory, which is on the same campus as Dhaka Hospital. The samples were transported in a specimen container containing two ice packs to maintain temperature.

Supplementary Document 3 - RNA extraction protocol from blood samples

Frozen Tempus Blood RNA tubes were thawed over ice. Four mL of the nine mL sample and reagent mix from each Tempus tube was mixed with 1.5 mL of Phosphate Buffer Saline, vortexed and centrifuged at 4000g for 20 minutes at 4°C. The supernatant was discarded, and the pellet was resuspended with 1 mL of TRIzol (ThermoFischer) or RNAiso Plus (Takara) followed by five minutes of incubation at room temperature. 0.3 mL of chloroform was added, shaken vigorously for 20 seconds, and incubated 2-3 minutes in ice before centrifuging 15 minutes at 12,000g and +4°C. The upper aqueous layer was transferred to a new tube. An equal volume of ice-cold isopropanol was added and incubated at -20°C for 1 hour, followed by centrifugation (12,000g, 4°C) to precipitate the RNA. The supernatant was discarded, and the pellet was washed with 1 mL of 70% cold ethanol and centrifuged (5 min, 10,500g, 4°C). The supernatant was discarded, the RNA pellet air dried (5-10 min), resuspended in 26 µL of DEPC-treated water through pipette mixing, and incubated at 56°C for 10 minutes. RNA was stored at -80°C or immediately used for cDNA synthesis.

Supplementary Document 4 – Cost per TB case identified

Test performance (Table S1) was evaluated in the two age groups using the Clinical Reference Standard (CRS) as the reference. The numbers in the positive fraction (TP + FP) and negative fraction (TN + FN) were subsequently disaggregated according to the diagnostic classification, based on the clinical case definition of intrathoracic tuberculosis in children, to ascertain the distribution of cases within the categories of 'TB confirmed,' 'TB unconfirmed,' or 'TB unlikely' categories.

To calculate the cost per TB case identified, two strategies were evaluated (Figure S3):

- The one-step strategy, where a single test is performed, and individuals with positive results proceed directly to confirmatory testing (Xpert).
- The two-step strategy is an iterative process. In the first step, all individuals are tested using TST or CXR. Those with positive results were referred for confirmatory testing. In the second step, individuals with negative CXR or TST results are tested using RISK6. Those with a positive RISK6 result are also referred for confirmatory testing. Individuals remaining negative are excluded from the TB diagnosis process and are not considered in the cost calculation for TB case identification.

In both evaluated strategies, the sensitivity, specificity, and prevalence data from the test results compared to the CRS were applied to a hypothetical cohort of 10,000 children with suspected pulmonary tuberculosis undergoing TB triage testing (Table S2). The numbers for each fraction (TP, FP, TN, FN) were calculated using the online GraphPad calculator (available at: https://www.graphpad.com/quickcalcs/clinTest2). The distribution across classification groups was derived by extrapolating the initial analysis data. The number of individuals requiring confirmatory testing and the number expected to test positive in the confirmation tests, for each strategy and test evaluated was determined.

GenXpert MTB/Rif Ultra (Xpert) was selected as the microbiological confirmation test for our simulation, due to its routine use in Bangladesh and WHO approval. Because individuals classified as “unconfirmed TB” who are known to be infected (TST positive) or who show signs of *Mycobacterium tuberculosis* infection (CXR interpretation) are not excluded and are even considered positive, Microbiological Reference Standard (MRS) was found to be more appropriate than the CRS as a reference standard for

evaluating confirmation test performance. We therefore evaluated the performance of the Xpert as confirmatory test, using MRS as the reference standard for induced sputum and stool samples (either individually or in combination) (Table S3). These performance metrics were applied to the positive fractions of the various strategies assessed, allowing the final number of microbiologically confirmed cases to be determined. In addition, in line with the latest WHO recommendations for TB identification tests in peripheral settings (3), we simulated a strategy using a hypothetical test that meets 75% sensitivity and 98% specificity threshold requirements in the WHO TPP. However, without any indication concerning the sample type, we decided to apply these thresholds to all the samples to be tested (Table S3).

Diagnostic costs (Table S4) were collected at the study site as well as documented in a recent study (2), adjusted for inflation in 2023 considering the inflation rates obtained from Bangladesh Bank.

It should be noted that we have not considered the skill level of the personnel required to conduct these tests, nor the cost of human resources. CXR requires a radiographer to generate the images and a radiologist doctor to interpret them. The TST requires a well-trained nurse to administer and analyze the result as well as two patient visits. RISK6 Assay requires a well-trained laboratory technician (in addition to a well-equipped laboratory). We therefore calculated the incremental cost per strategy and sample type implemented:

1- For triaging (Table S5): we considered the cost of a medical consultation, the tests performed, and sampling, if necessary, then multiplied the total by 10,000, representing the number of individuals in the theoretical cohort presenting with presumptive TB.

2- For confirmation (Table S6): we considered the cost of a medical consultation, one or two Xpert cartridges and the cost of sampling (induced sputum and/or stool), then multiplied the total by the number of presumptive TB individuals

The incremental cost (Table S7) corresponds to the sum of the costs calculated for triaging and confirmation.

We then divided the incremental cost by the number of confirmed TB cases identified after confirmation testing to obtain the cost per identified TB case.

Supplementary Document 5 - Comparison of RISK6 performance to discriminate between confirmed TB cases and unconfirmed TB cases using TB treatment as reference standard

Figure S4 and Table S8

Supplementary Document 6 - Evaluation of test performance using MRS as reference standard

Table S9

Supplementary Document 7 - Evaluation of Xpert Ultra performance using CRS as reference standard

Table S10

Bibliography

1. Rahman SMM, et al. Evaluation of Xpert MTB/RIF assay for detection of Mycobacterium tuberculosis in stool samples of adults with pulmonary tuberculosis. PloS One. 2018;13(9):e0203063.

2. Hasan MdZ, et al. Costs of services and funding gap of the Bangladesh National Tuberculosis Control Programme 2016–2022: An ingredient based approach. PLOS ONE. 2023;18(6):e0286560. Available from: https://www.ncbi.nlm.nih.gov/pmc/articles/PMC10237497/

3. Target product profile for tuberculosis diagnosis and detection of drug resistance. Available from: https://www.who.int/publications/i/item/9789240097698
